# *Sumatrella chelonica* gen. n., sp. n., a new remarkable genus and species from Indonesia, Sumatra (Acari, Uropodina, Oplitidae)

**DOI:** 10.3897/zookeys.484.8836

**Published:** 2015-02-25

**Authors:** Jenő Kontschán

**Affiliations:** 1 Plant Protection Institute, Centre for Agricultural Research, Hungarian Academy of Sciences, H-1525 Budapest, P.O. Box 102, Hungary

**Keywords:** South-East Asia, taxonomy, turtle mites

## Abstract

A new genus *Sumatrella* **gen. n.** is described and illustrated based on the new species *Sumatrella chelonica* **sp. n.** collected in Sumatra, Indonesia. The new genus belongs to the family Oplitidae based on its hypertrichous internal malae and the absence of strongly sclerotized structures on the dorsal shield. The new genus is closely related to the genus *Chelonuropoda* Sellnick, 1954 but the transverse furrow on ventral idiosoma close to coxae IV and the strongly sclerotized C-shaped dorsal line are missing in the new genus. These characters can be found in species of *Chelonuropoda*.

## Introduction

The Uropodina mites are one of the well-characterized members of the soil mite fauna. They can be found with a high diversity in tropical regions (Lindquist et al. 2009), but currently only 10% of the known species are from this region (Vázquez and Klompen 2007).

The family Oplitidae (Lindquist et al. 2009, Beaulieu et al. 2011) is a very district group among the Uropodina, which possesses several specific characters. The internal malae is subdivided into several branches bearing pilose margins, the hypostomal setae are not situated in a longitudinal row, and the dorsal shield does not bear strongly sclerotized structures. Its members can often be found in ant nests (Mašán 2001). This family was previously treated as one genus, and divided into several species groups in previous systems (Wiśniewski and Hirschmann 1993). Recently it was elevated to family level [Lindquist et al. (2009), Beaulieu et al. (2011)] and is divided into further genera (e.g. Kontschán 2010, Kontschán and Starý 2012).

In 2014, some days were spent in the Arachnida collection of Natural History Museum in Geneva, where I found several specimens of a very unusual oplitid species, described here as a new genus and new species.

## Material and methods

Specimens of this unusual species were cleared in lactic acid, investigated on half-covered deep slides and illustrations were made with the aid of a drawing tube. Photographs were taken with a Nikon CoolPix900 digital camera. All specimens are stored in ethanol and deposited in the Natural History Museum in Geneva (NHMG). All measurements are given in micrometres (μm).

## Taxonomic

### Sumatrella gen. n.

Taxon classificationAnimaliaMesostigmataOplitidae

http://zoobank.org/360C7C81-9BA6-4CEC-B92A-9F02B5E06768

#### Diagnosis.

Idiosoma small, oval, posterior margin rounded and very convex. All part of marginal shield wide and fused anteriorly to dorsal shield. Dorsal and ventral setae smooth and needle-like. Genital shield of female octagonal, without sculptural pattern and anterior process. Dorsal and marginal shields neotrichous. Corniculi horn-like, internal malae with several long branches. Hypostomal setae h3 longer than others, h2 situated outside the longitudinal row h1–h4 and shorter than others. Tritosternum with narrow basis, laciniae divided into two short and two long pilose branches. Epistome hemispherical and marginally pilose. Leg I without claw, trochanters II-IV with a triangular process.

#### Type species.

*Sumatrella chelonica* sp. n.

#### Etymology.

The name of the new genus refers to the name of island where the specimens were collected. Gender feminine.

#### Systematic notes.

On the basis of the shape of internal malae (divided into pilose branches), the absence of the T-shaped dorsal setae and the hypostomal setae h2 position lateral to row h1–h4, I refer this genus to Oplitidae. Recently several genera and species groups have been recognized in this family (Kontschán 2010, Kontschán and Starý 2012, Wiśniewski and Hirschmann 1993), but the new genus differs from the others on the basis of the very convex idiosoma, the octagonal genital shield and the wide marginal shield. Only the genus *Chelonuropoda* Sellnick, 1954 shares this combination of character states with the new genus (i.e. very convex idiosoma and wide marginal shield) but the former differs in several characters, the most important of which are summarized in Table 1.

**Table 1.** The distinguishing characteristics between *Chelonuropoda* Sellnick, 1954 and *Sumatrella* gen. n.

	*Chelonuropoda*	*Sumatrella*
Length of idiosoma	1000<	600>
Width of marginal shield	only on anterior area	on all area
C-shaped strongly sclerotized dorsal lines	present	absent
Transverse furrow near coxae IV on ventral idiosoma	present	absent
Shape of female genital shield	linguliform	octagonal
Shape of peritreme	long, hook-like, mushroom-like or R-shaped	short, C-shaped
Epistome	triangular	hemispherical
Triangular process on trochanters of legs II–IV	absent	present
Claws on leg I	present	absent

### Sumatrella chelonica sp. n.

Taxon classificationAnimaliaMesostigmataOplitidae

http://zoobank.org/F7450850-E63B-4B07-B18F-346699ACF37F

Figs 1–4 , 5–8 , 9–11 , 12–16 , 17–22

#### Material examined.

*Holotype*. Female. Indonesia, Sumatra, West Sumatra Province, primary forest at buttom of Haran Canyon, near Echo Point, N of Pavakumbah, 0°06'21"S, 100°39'50"E, 500m, 8.VI.2006. leg. A. Schulz. *Paratypes*. Three females from Indonesia, Sumatra, West Sumatra Province, primary forest at buttom of Haran Canyon, near Echo Point, N of Pavakumbah, 0°06'21"S, 100°39'50"E, 500m, 8.VI.2006. leg. A. Schulz and 7 females from Indonesia, Sumatra, West Sumatra Province, distributed primary forest near road Lubuksikaping Bonjol, *ca.* 10 km S of Lubuksikaping, 0°03'16"N, 100°12'33"E, 500m, 12.VI.2006. leg. A. Schulz.

#### Diagnosis.

As for the genus.

#### Description of the females.

Length of idiosoma 560–580 µm, width 470–510 µm, height 560–570 µm (n=11). Shape oval, posterior margin rounded and dorsally extremely domed (Figs 18–19). Color reddish brown.

**Figures 1–4.**
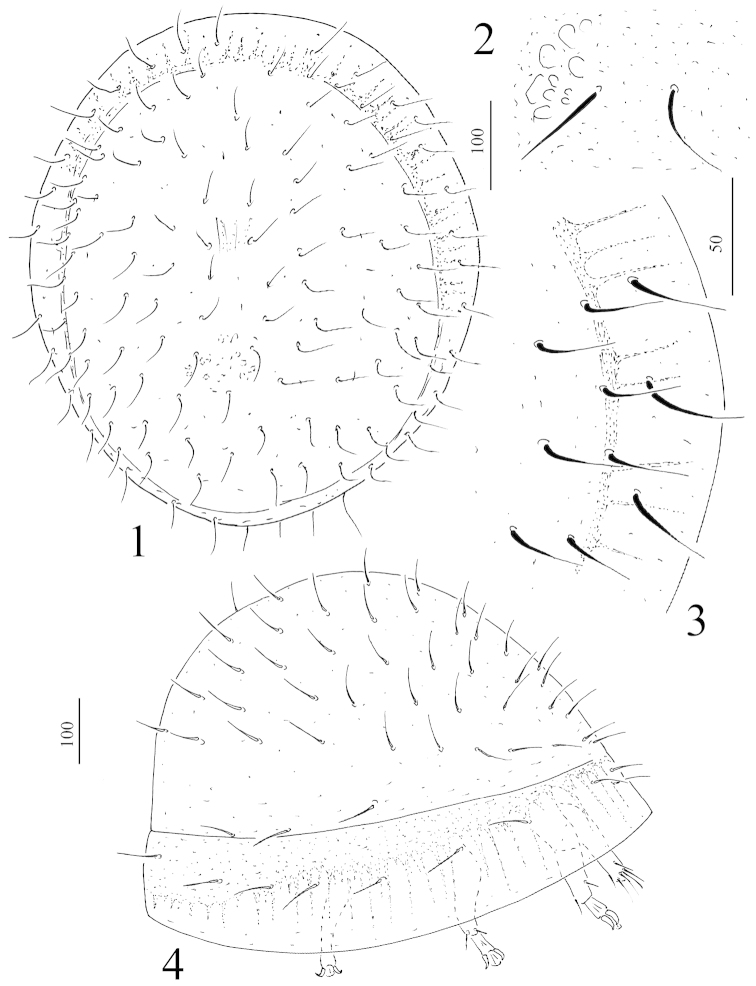
*Sumatrella chelonica* gen. n., sp. n., female, holotype: **1** body in dorsal view **2** setae on dorsal shield **3** setae on marginal shield **4** body in lateral view. Scale bars in micrometers.

*Dorsal idiosoma* (Figure 1): Dorsal and marginal shields fused apically. Dorsal shield neotrichous, all dorsal setae smooth and needle like (*ca.* 32–44 µm) (Figure 2). Surface of dorsal shield smooth, only some muscle scars can be seen at level of coxae IV. Marginal shield very wide (Figure 4) with darker and spine–like patterns on inner margins, setae on marginal shield similar in shape and length to dorsal setae (Fig. 4).

**Figures 5–8.**
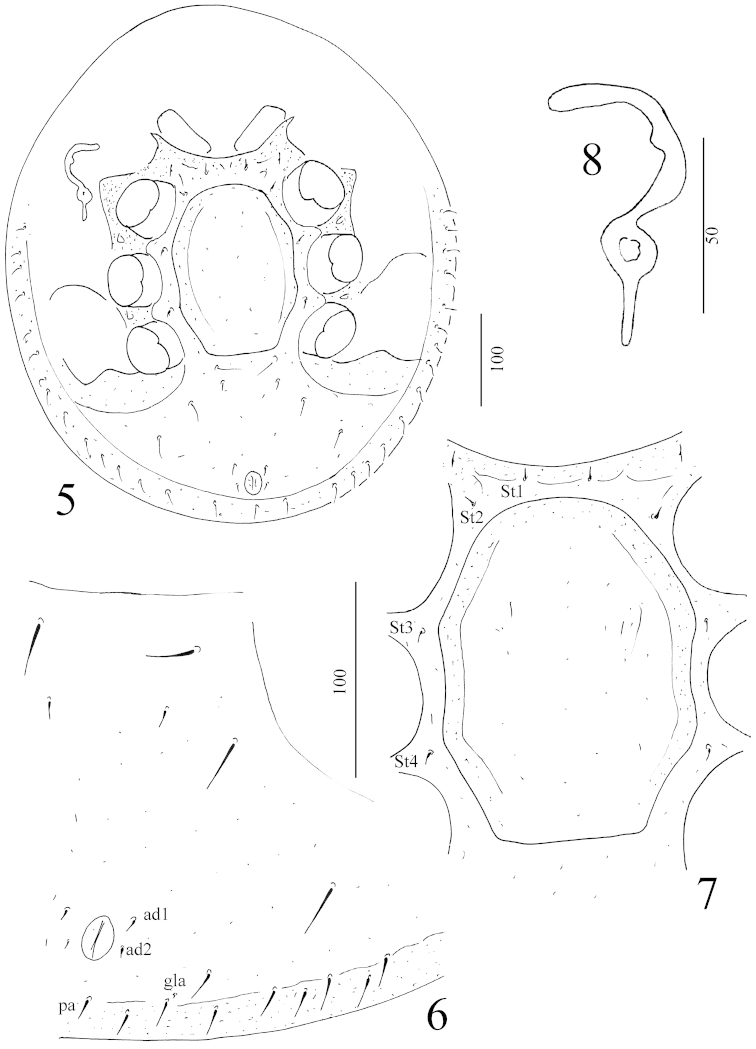
*Sumatrella chelonica* gen. n., sp. n., female, holotype: **5** body in ventral view **6** anal and ventral regions **7** intercoxal area **8** peritreme. Scale bars in micrometers.

**Figures 9–11.**
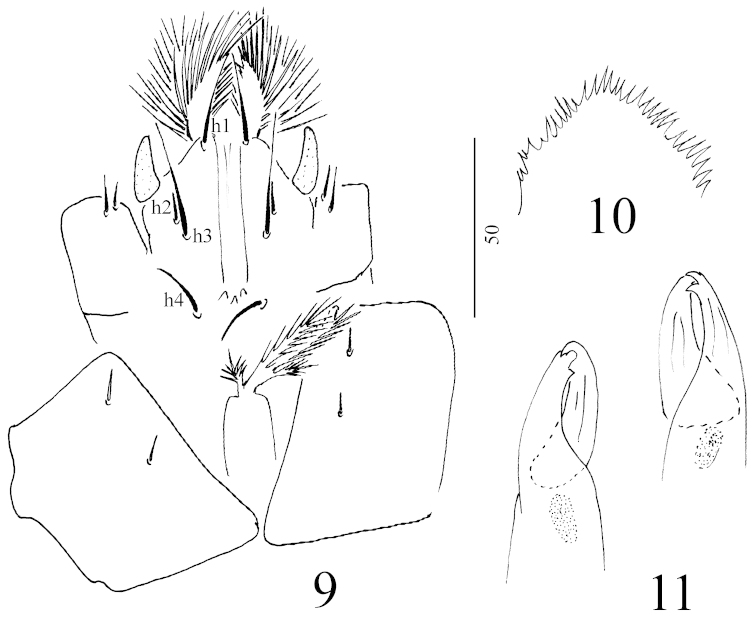
*Sumatrella chelonica* gen. n., sp. n., female, holotype: **9** ventral view of tritosternum, gnathosoma and coxae I **10** epistome **11** chelicerae. Scale bars in micrometers.

*Ventral idiosoma* (Figure 5): Tritosternum with narrow, quadrangular basis; laciniae with two short and two long pilose branches (Figure 9). Sternal shield without ornamentation, four pairs of sternal setae smooth, short (*ca.* 6–7 µm) and needle-like. St1 situated near anterior margin of sternal shield, St2 at level of anterior margin of genital shield, St3 at level of anterior margin of coxae III, St4 at level of posterior margin of coxae III. One pair of lyriform fissure situated close to anterior margin of sternal shield. Three pairs of longer (*ca.* 25–30 µm) and one pair of shorter (*ca.* 10–11 µm) ventral setae situated, all ventral setae smooth and needle-like. Two pairs of adanal setae short (*ca.* 5–6µm) smooth and needle-like, postanal seta smooth, needle-like and long (*ca.* 14–15 µm). Second setae from postanal seta associated with a setae-like sensory organ. Margins of ventral idiosoma bearing numerous smooth and needle-like setae (*ca.* 14–17 µm). Surface of ventral shield without ornamentation (Figure 6). Genital shield octagonal, *ca.* 180 µm long and *ca.* 130 µm wide, without sculptural pattern and anterior process. Stigmata situated between coxae II and III. Prestigmatic part of peritremes C-shaped with a very short central branch, poststigmatic part short and straight (Figure 8). Pedofossae deep, their surface smooth, separated furrows for tarsi IV absent.

*Gnathosoma* (Figure 9): Corniculi horn-like, internal malae longer than corniculi and divided into several pilose branches. All hypostomal setae smooth and needle-like, h1 (*ca.* 25–27 µm) situated near anterior margin of gnathosoma, h2 very short (*ca.* 9–10 µm) and situated close to h3 and placed lateral to h1-h4 row. Setae h3 long (*ca.* 33–35 µm), h4 shorter (*ca.* 16–17 µm). Three ventral denticles situated on central part of ventral gnathosoma at level of h4.

**Figures 12–16.**
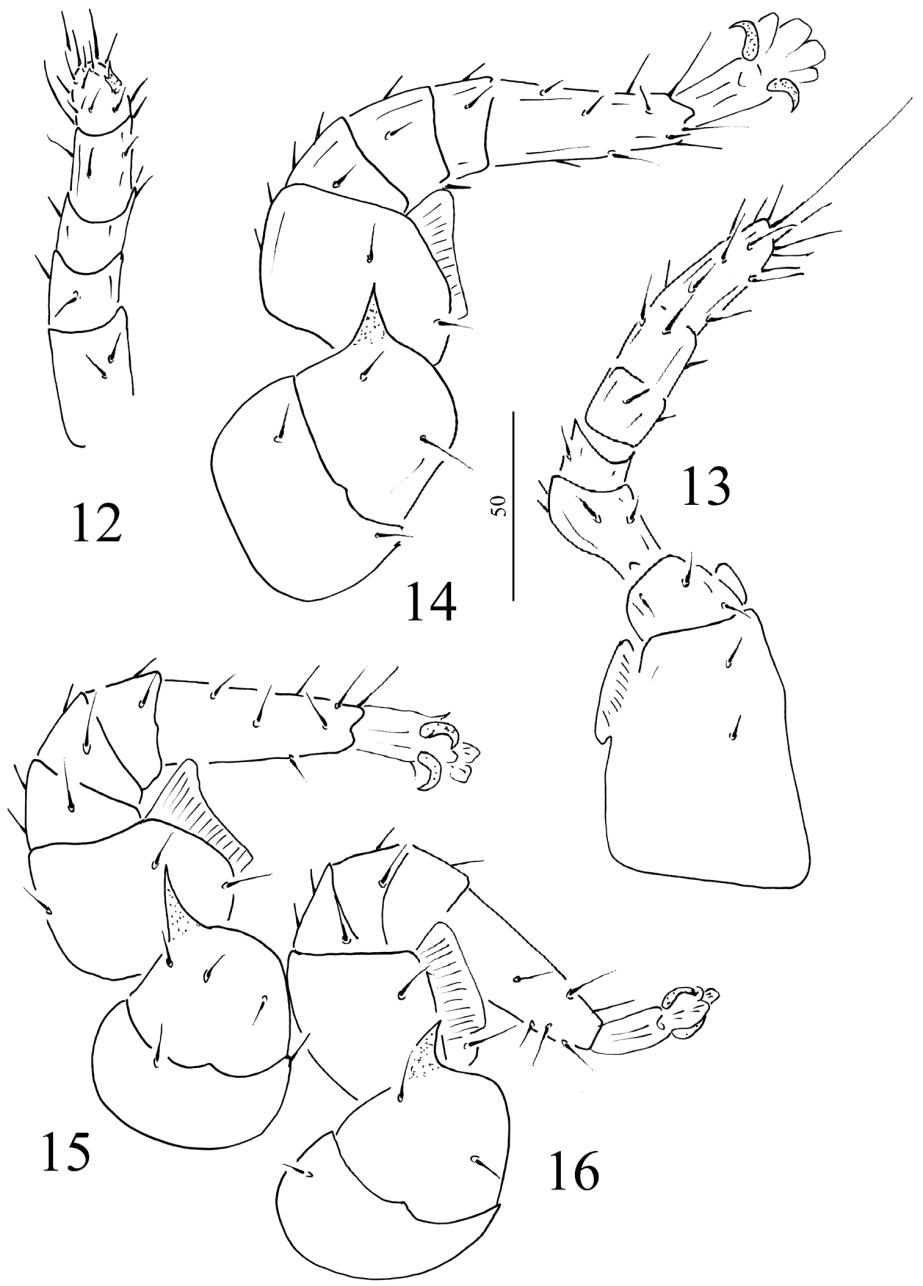
*Sumatrella chelonica* gen. n., sp. n., female, holotype: **12** ventral view of palp **13** ventral view of leg I **14** ventral view of leg II **15** ventral view of leg III **16** ventral view of leg IV. Scale bars in micrometers.

All setae on palp smooth and needle-like (Figure 12). Epistome hemispherical and marginally pilose (Figure 10), chelicerae with one teeth on movable and fixed digits, internal sclerotized node present (Figure 11). Scale bars in micrometers.

**Figures 17–22.**
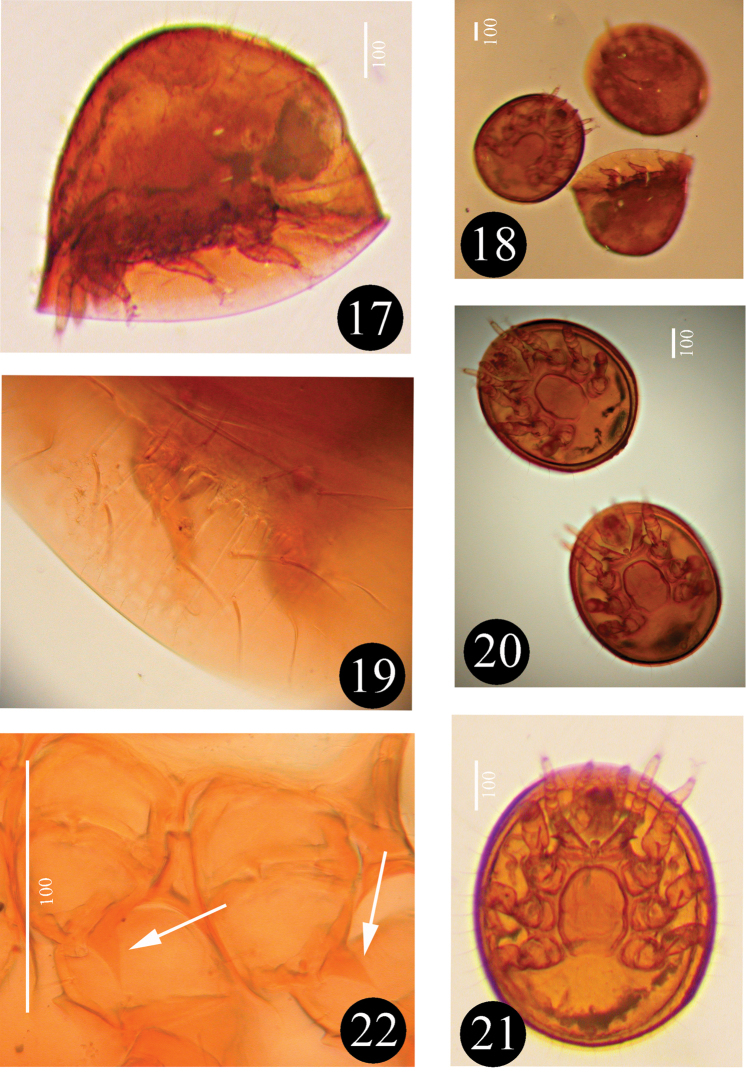
Photos about *Sumatrella chelonica* gen. n., sp. n., female: **17** body in lateral view in holotype **18** bodies in lateral, ventral and dorsal views in paratypes **19** marginal shield and setae in holotype **20** bodies in ventral view of paratypes **21** body in ventral view of holotype III **22** triangular processes on trochanters of legs II-III in holotype (arrows show the processes).

*Legs* (Figures 15–18): Claws absent at the tip of the ambulacral prolongation of leg I. Flap-like prolongations placed on femora II–IV and an unusual triangular process situated on trochantes II-IV.

Male, nymph and larva are unknown.

#### Etymology.

The name of the new species refers to the raised shape of the mite body which is reminiscent of a turtle.

## Zoogeographical notes

Species of the probably closely related genus *Chelonuropoda* Sellnick, 1954 occur in South America and the Afrotropical region; a distribution pattern which has been named ‘Amphiatlantic’ (Kontschán and Starý 2012). Based on zoogeography, the genus *Chelonuropoda* must have originated during a geological period when Africa and South America were still connected to each other; i.e. prior to the Upper Cretaceous. The new genus occurring on Sumatra Island is not situated on a Gondwanan fragment. Therefore we can consider two hypotheses about its distribution: either Sumatra was colonized by the new genus by other dispersal means, or the similarities in morphology are the result of parallel evolution and are examples of homoplasy.

## Supplementary Material

XML Treatment for Sumatrella

XML Treatment for Sumatrella chelonica
